# Quality controlled, reliable groundwater level data with corresponding specific yield over India

**DOI:** 10.1038/s41597-025-05899-5

**Published:** 2025-10-01

**Authors:** Satish Kumar Kuruva, Maya Raghunath Suryawanshi, Amin Shakya, Chethan VA, Balaram Shaw, Vandana Sukumaran, Retinder Kour, Aayushi Kochar, Shard Chander, Bhaskar R. Nikam, Nagesh Kumar Dasika, Bramha Dutt Vishwakarma

**Affiliations:** 1 Interdisciplinary Centre for Water Research, Indian Institute of Science, Bengaluru, India; 2 Faculty of Geo-Information Science and Earth Observation, University of Twente, Enschede, Netherlands; 3 Land Hydrology Division, Space Applications Centre, Indian Space Research Organisation, Ahmedabad, India; 4 Indian Institute of Remote Sensing, Indian Space Research Organisation, Dehradun, India; 5 Department of Civil Engineering, Indian Institute of Science, Bengaluru, India; 6 Centre for Earth Sciences, Indian Institute of Science, Bengaluru, India

**Keywords:** Hydrology, Water resources

## Abstract

Groundwater is a vital resource for domestic, agricultural, and industrial use, with its demand growing due to population growth and climate change. Several studies have identified groundwater depleting in India at unsustainable rate over North-west part, but a contrasting trend is observed in the southern India. To better study groundwater dynamics quality-controlled and reliable well data is essential, which is missing. Here we process seasonal groundwater levels from 32,299 wells across India to obtain reliable well data and provide respective specific yields. Initially, wells with no data and negative values are removed. Later three-sigma method is imposed on each well to eliminate outliers. Finally, wells with at least two values per year, with no value repeating more than twice consecutively, are retained, resulting in 2,759 reliable wells. We used vectorization-based method to classify aquifer types and estimated specific yields based on hydrogeological map. We also provide open access to data and scripts so that researchers can study groundwater variations, compare GRACE and model-based groundwater estimates against *in-situ* well data.

## Background & Summary

Groundwater is a vital component of the hydrological cycle that serves as a primary source of fresh water for drinking, irrigation, and industrial use^1^. As global population rises and climate patterns shift, groundwater’s role is expected to grow, particularly as surface water becomes less reliable. By 2050, an additional 2 billion people will need to be fed, driving increased demand for agricultural land and water resources^2^. In India, for instance, a whooping 160 billion cubic meters of groundwater is extracted annually for irrigation, accounting for 35% of the nation’s total water demand^3^. With climate change intensifying droughts and altering regional recharge dynamics, groundwater is becoming an increasingly vital indicator of food security in a changing climate^4–6^.

The use of groundwater and its eventual depletion is a complex issue with far-reaching consequences, impacting not just water supply but also broader environmental and societal systems. Over-extraction of groundwater leads to land subsidence, decreased baseflow, coastal saltwater intrusion and intensifying the political conflict across transboundary aquifers^7–11^. Therefore, it is crucial to monitor and understand the availability of groundwater across the aquifer systems, especially as they are increasingly exploited by human activities. Most of our understanding of groundwater usage and availability is derived from *in-situ* measurements, which are difficult to collect and maintain as a long term records. Techniques like groundwater well monitoring face additional complications due to the heterogeneous nature of geology, soil type, aquifer structure, and specific yield, all of which vary across regions^12,13^. Even in areas where *in-situ* data is more abundant, challenges arise in aggregating, integrating, and interpreting these data effectively. As a result, there is a critical need for high-quality, reliable groundwater records that can improve our understanding of groundwater dynamics. Such records are essential for preventing overuse and developing sustainable water policies.

India is one of the largest consumers of groundwater across the globe owing to nations large population and intensive agricultural activities^14,15^. Indeed, domestic water use and irrigation account for about 50–80% and 45–50% of total available groundwater, respectively. Concurrently it is observed from satellite as well as *in-situ* data that the groundwater, which drives Indian agro-economy, is declining with alarming rate due to increased irrigation^16^. Satellite-based observations from the Gravity Recovery And Climate Experiment (GRACE), which exclusively provides information about groundwater storage, needs specific yield and quality-controlled groundwater levels for its validation. The existing  Central Ground Water Board (CGWB)  GroundWater Level (GWL) dataset is available at seasonal scale with numerous data gaps and limitations to extract. Therefore, in this study, we apply rigorous filtering methods to seasonal groundwater level data, providing a set of quality-controlled and reliable groundwater records for India between 2000 to 2022. This effort aims to offer valuable data to the community and support better water resource management practices.

In addition to that, we provide specific yield value at individual well locations by processing available hydrogeological map from Bhanja *et al*.^17^. The Specific Yield (S_y_) is the ratio of the volume of water that drains from a saturated rock owing to the attraction of gravity to the total volume of the rock^18^. The ratio is usually expressed as a percentage indicating the amount of water released from an unconfined aquifer. The S_y_ value is not conclusive because the amount of water that will drain by gravity varies on several factors, including temperature, drainage duration, the water’s mineral composition, and other physical attributes of the rock or soil under consideration. However, S_y_ values are highly helpful in hydrologic investigations because they provide a practical way for hydrologists to determine the water-yielding capacity of subsurface materials. Furthermore, they are related to GRACE-based  Equivalent Water Height (EWH) values via a simple linear relation (Eq. 1).

1 $$\triangle S=\triangle H\times {S}_{y}$$

Where, $\triangle {S}$ represents groundwater storage changes, and $\triangle {H}$ represents groundwater level changes.

Various field and laboratory methods are available to determine the S_y_ of a material. Even with the availability of numerous laboratory and field experiments, there is still a certain degree of uncertainty in calculating the S_y_ because laboratory samples may be disturbed, while in field tests, controlling and measuring hydrogeological variables is challenging, often resulting in inaccurate estimates. Since there is currently no commonly recognized method for estimating S_y_, as highlighted by Zhang *et al*.^19^ and Dietrich *et al*.^20^, determining a suitable S_y_ value in practice remains a significant difficulty. Therefore, the present study estimates S_y_ at each well location which will help in gaining a deeper understanding in the model accuracy, ultimately leading to more reliable predictions of groundwater resources and water availability across diverse hydrological conditions, both at regional and global scales.

Hence there are two key objectives: (1) Filtering the available groundwater level dataset to generate quality-controlled and reliable records at seasonal scales from 2000 to 2022 across India, and (2) Estimating the point based S_y_ at each well location throughout the country.

## Methods

### Groundwater level measurements

Central Ground Water Board (CGWB), India measures the GWLs four times a year during January, May, August and November manually which are available from India Water Resources Information System (https://indiawris.gov.in/wris/#/). The quarterly water level measurements depict GWL scenarios during various seasons. For example, post-monsoon water levels are measured in January and November, pre-monsoon water levels are measured in May, and monsoon-time water levels are measured in August. The CGWB started the monitoring of GWLs in the year 1969 and as of 30.04.2023, a network of 25,437 observation wells is being monitored all over India^21^. However, accessing this data for bulk analysis is cumbersome, as users must download the time-series data district-wise, one district at a time. The observation wells are divided into seven categories namely dug well, bore well, tube well, dug cum bore well, slim hole, miscellaneous (unknown) and piezometer. In the present study, we used GWL observations available from more than 32,000 wells. However, data from 2,759 wells with long-term records were chosen for investigation following quality checks and postprocessing. The data is processed to ensure temporal continuity, meaning that for every year over the whole study area, at least two of the four seasonal data should be available. The period considered for the present work is from 2000 to 2022. The scripts are also provided to help readers or users to apply similar filtering and selection criteria to extract reliable quality controlled well data for another period or over another region.

### Filtering criteria for groundwater level measurements

The manual GWL measurements from CGWB from 2000 to 2022 are taken into consideration in this study. The units of GWLs are meters below ground level (m bgl). As indicated in Table 1, there are 32299 wells in total, which are separated into the following categories: dug well (20265), bore well (4589), tube well (4246), miscellaneous/unknown (2239), piezometer (935), dug cum bore well (24), and slim hole (1). To obtain reliable well observation data covering the entire study area, filtering criteria are applied on the well data.

**Table 1:** Well classification and its count for India.

S. No	Type of Well	Well Count
1	Dug Well	20265
2	Bore Well	4589
3	Tube Well	4246
4	Miscellaneous/unknown	2239
5	Piezometer	935
6	Dug cum Bore Well	24
7	Slim Hole	1
	**All India Well count**	**32229**

Five types of filtering criteria are applied to the dataset. First, wells with no data are removed. Second, wells with negative values are removed. Third, three-sigma method is imposed on each well to eliminate outliers. Fourth, wells with at least two values per year are retained. Finally, wells where GWLs repeat consecutively more than twice are removed. Therefore, the 2,759 consistent wells identified through these filtering criteria are extracted for the entire country. Details of the filtering criteria are provided in Table 2.

**Table 2:** Filtering criteria applied for the well observations.

S. No	Filtering criteria (Jan 2000 to Dec 2022)	Well Count
1	All India well data	32299
2	Remove wells with no data	32094
3	Remove wells with negative values	31169
4	Remove outlier within a well	31169
5	Minimum two GWL values per year	2759

### Hydrogeological map of India

**Fig. 1:**
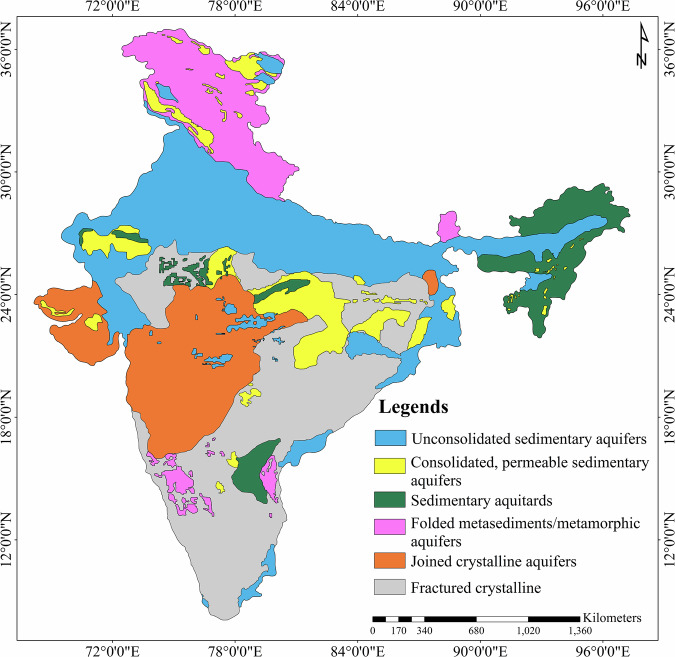
Hydrogeological map of India (Source: Bhanja *et al*.^17^).

The hydrogeological map of India (Fig. 1, Bhanja *et al*.^17^) is used to obtained S_y_ values at the filtered well locations. The map indicates that whole Indian aquifer system is divided into six different hydrogeological units: Unconsolidated sedimentary aquifers, consolidated, permeable sedimentary aquifers, sedimentary aquitards, folded metasediments/metamorphic, joined crystalline, and fractured crystalline. The numerical values assigned to those aquifer types are mentioned in Table 3.

**Table 3:** Specific yield values for varying hydrogeological setting shown Fig. 1 (Source: Bhanja *et al*.^17^).

S. No	Hydrogeology	S_y_ range	Mean S_y_
1	Unconsolidated sedimentary	0.06 to 0.20	0.130
2	Consolidated, permeable sedimentary	0 to 0.08	0.043
3	Sedimentary aquitards	0 to 0.03	0.018
4	Folded metasediments/metamorphics	0 to 0.03	0.018
5	Jointed crystalline	0.01 to 0.03	0.020
6	Fractured crystalline	0 to 0.04	0.023

### Specific yield extraction using vectorization-based method

To estimate S_y_ values at the filtered well locations, the hydrogeological map of India from Bhanja *et al*.^17^, available in Portable Network Graphics (PNG) format, was georeferenced to align with spatial coordinates in Quantum Geographic Information System (QGIS) software. Six hydrogeological units were then manually digitized (Fig. 1) from the georeferenced map to create a vector shapefile. The corresponding S_y_ values for each hydrogeological unit, as reported by Bhanja *et al*.^17^, were assigned to the attribute table of the shapefile. This vector layer was subsequently converted into a raster format to facilitate spatial analysis. The filtered well locations, initially provided in a.csv file, were converted into a point shapefile and overlaid on the raster layer. S_y_ values were then extracted from the raster at each well location using “Sample raster values” tool under “Raster analysis”.

### Dataset used for validation of specific yield

GRACE satellite derived GroundWater Storage Changes (GWSC_GRACE_) are used to validate estimated S_y._ To derive GWSC_GRACE_, the monthly total water storage changes from GRACE Jet Propulsion Laboratory (JPL) RL06 mascon product are used (https://grace.jpl.nasa.gov/). Soil moisture storage changes, obtained from the Copernicus Climate Change Service (C3S) (https://cds.climate.copernicus.eu/) are subtracted from the total water storage to isolate GWSC_GRACE_. Next, quality controlled GWL  Changes (GWLC) are converted to *in-situ* GWSC (GWSC_insitu_) by multiplying with estimated S_y_ as shown in Eq. 2.

2 $${GWS}{C}_{{insitu}}={GWLC}\times {S}_{y}$$

Thereafter, GWSC_insitu_ are compared with GWSC_GRACE_ using correlation coefficient (r) (Eq. 3) and  Root Mean Square Error (RMSE) (Eq. 4) for the period 2002 to 2022.

3 $$r=\frac{\sum ({x}_{i}-\bar{x})({y}_{i}-\bar{y})}{\sqrt{\sum {({x}_{i}-\bar{x})}^{2}\sum {({y}_{i}-\bar{y})}^{2}}}$$

4 $$RMSE=\sqrt{\frac{{\sum }_{i=1}^{n}{({x}_{i}-{y}_{i})}^{2}}{n}}$$

Where ${x}_{i}={GWS}{C}_{{GRACE}}$, ${y}_{i}={GWS}{C}_{{insitu}}$, $\bar{x}$ and $\bar{y}$ denote the mean values of the respective datasets, n represents total number of observations.

Also note that the native resolution of GRACE JPL mascon is ~3° × 3°. However, quality controlled GWLs is a point data. Thus, in order to facilitate this comparison, wells within a given mascon are averaged and compared with GWSC_GRACE_.

## Data Records

The seasonal GWLs are obtained from CGWB (https://indiawris.gov.in/wris/#/). The hydrogeological map is obtained from Bhanja *et al*.^17^. Using these datasets S_y_ values are extracted for each well using vectorization-based method, which are available to users on figshare^22^. The final output CSV file is named as “CGWB_India_qualiy_controlled_GWLs_ref_sy_2000_2022.csv” with 2760 rows × 113 columns.

This file contains quality controlled GWL data from 2,759 stations (wells) across India. Each row represents a unique station (well) and includes metadata such as Station Code, Station Name, Station Type, Agency Name, State, District, Tehsil, Data Acquisition method, Block, Village, Latitude, Longitude, Data Available From, Latest Data Available, Type of Well, Aquifer Type, Well Depth, Data Type Code, Data Type Description, and Unit. Following the metadata, the file includes seasonal (Jan, May, Aug, Nov) GWLs (in m bgl) organized by specific months and years (e.g., Jan-00, May-00), covering from 2000 to 2022. The last column named as “Reference_Sy” represents the S_y_ value estimated in the present study using vectorization-based method.

## Technical Validation

### Groundwater well selection process

**Fig. 2:**
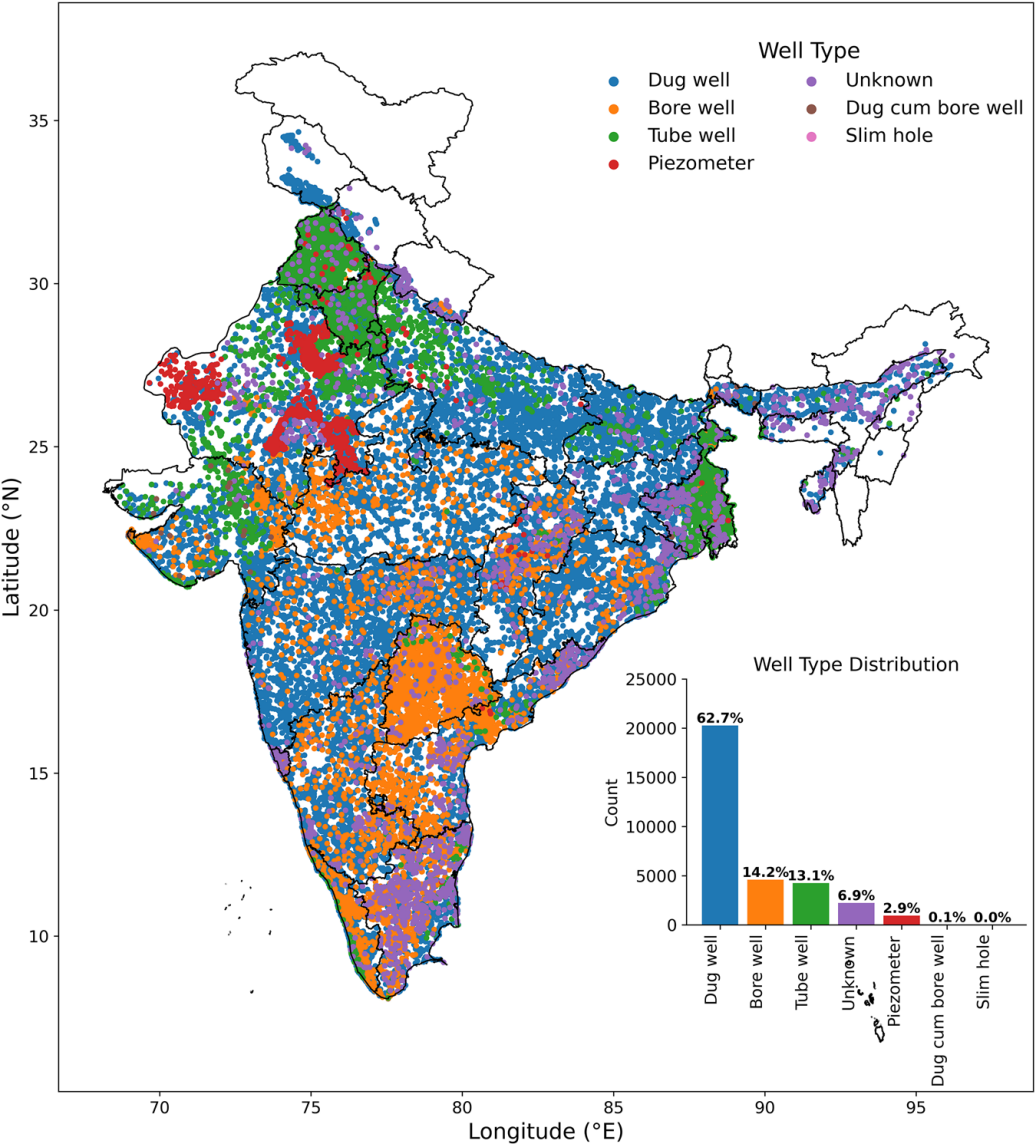
Well type distribution over India.

Manual GWLs from 2000 to 2022 are downloaded from CGWB at seasonal scale. Figure 2 displays the spatial distribution of seven distinct well types. Dug wells make up the largest portion of these well types, accounting for over 60% of all wells, followed by bore wells (14.2%) and tube wells (13.1%). The dug wells are dispersed equally throughout India apart from few states like Ladakh, Jammu and Kashmir, Himachal Pradesh, Uttarakhand and Assam due to unavailability of GWL datasets. The bore wells are widely distributed in the southern Indian states like Telangana, Andhra Pradesh, Karnataka, Tamil Nadu and Kerala as well as in a few central Indian states like Maharashtra, Madhya Pradesh etc. High density of tube wells is available in the states of Punjab, Haryana, West Bengal and even distribution of tube wells can be observed in the states of Rajasthan, Gujarat and along Eastern coastline of India. The piezometer wells are mainly present in Rajasthan. With 32,299 groundwater wells, India has a huge network of GWLs that helps in understanding GWL usage, distribution and extraction at both spatial and temporal dimensions.

**Fig. 3:**
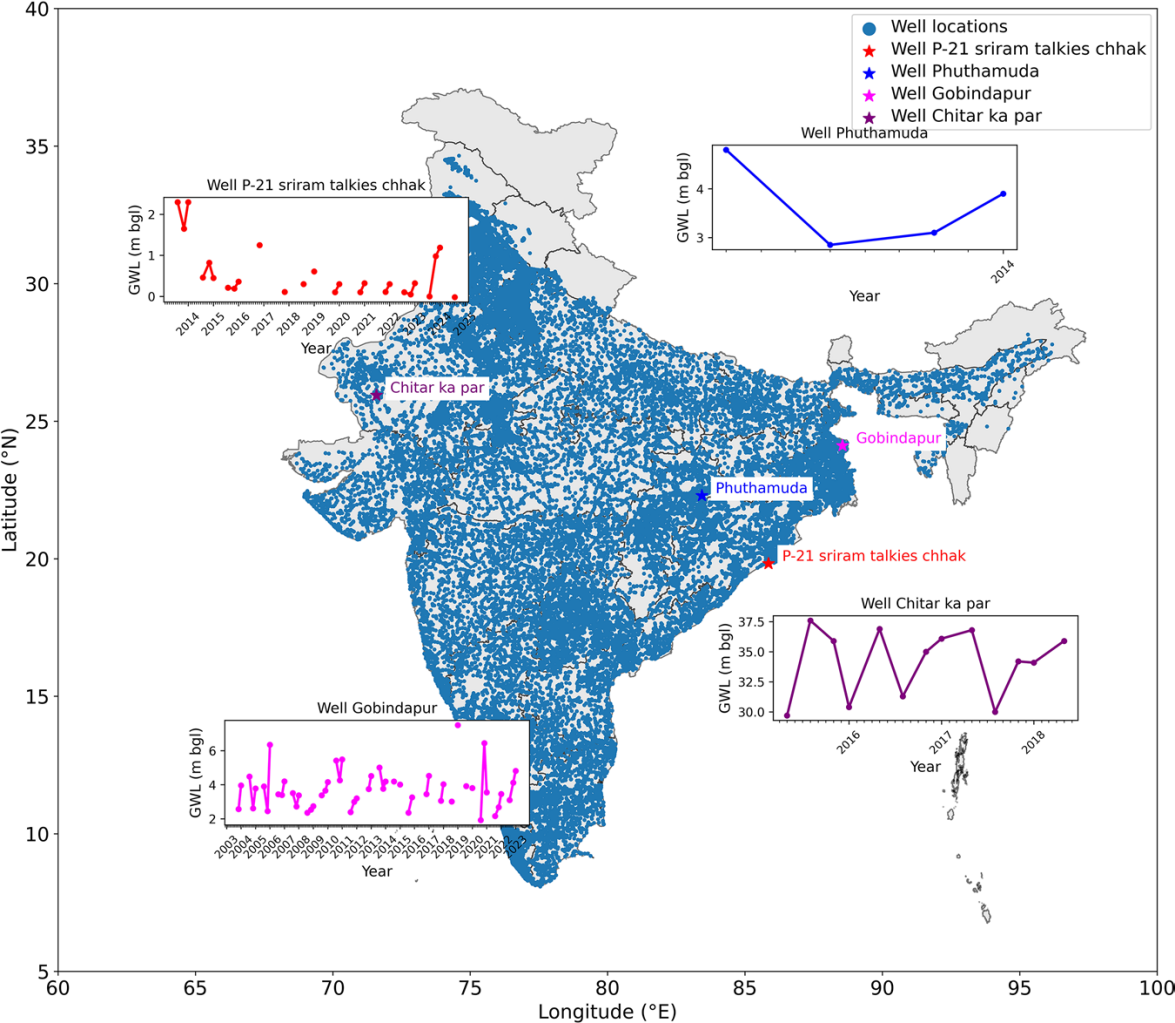
GWL variations for unfiltered wells.

Figure 3 displays the distribution of all well types without using the filtered criteria. Repetitive levels and numerous data gaps are to be expected because the data is not filtered. The use of unprocessed GWLs causes volatility in yield values as they are sensitive to time and space. As a result, the current analysis used the filtered GWLs for the extraction of S_y_ at each well location. A time series of four randomly chosen wells is shown in Fig. 3, and it is evident that there are missing data gaps in three wells between 2000 to 2022 (P-21 sriram talkies chhak, Puthamuda, Gobindapur, Chitar ka par).

**Fig. 4:**
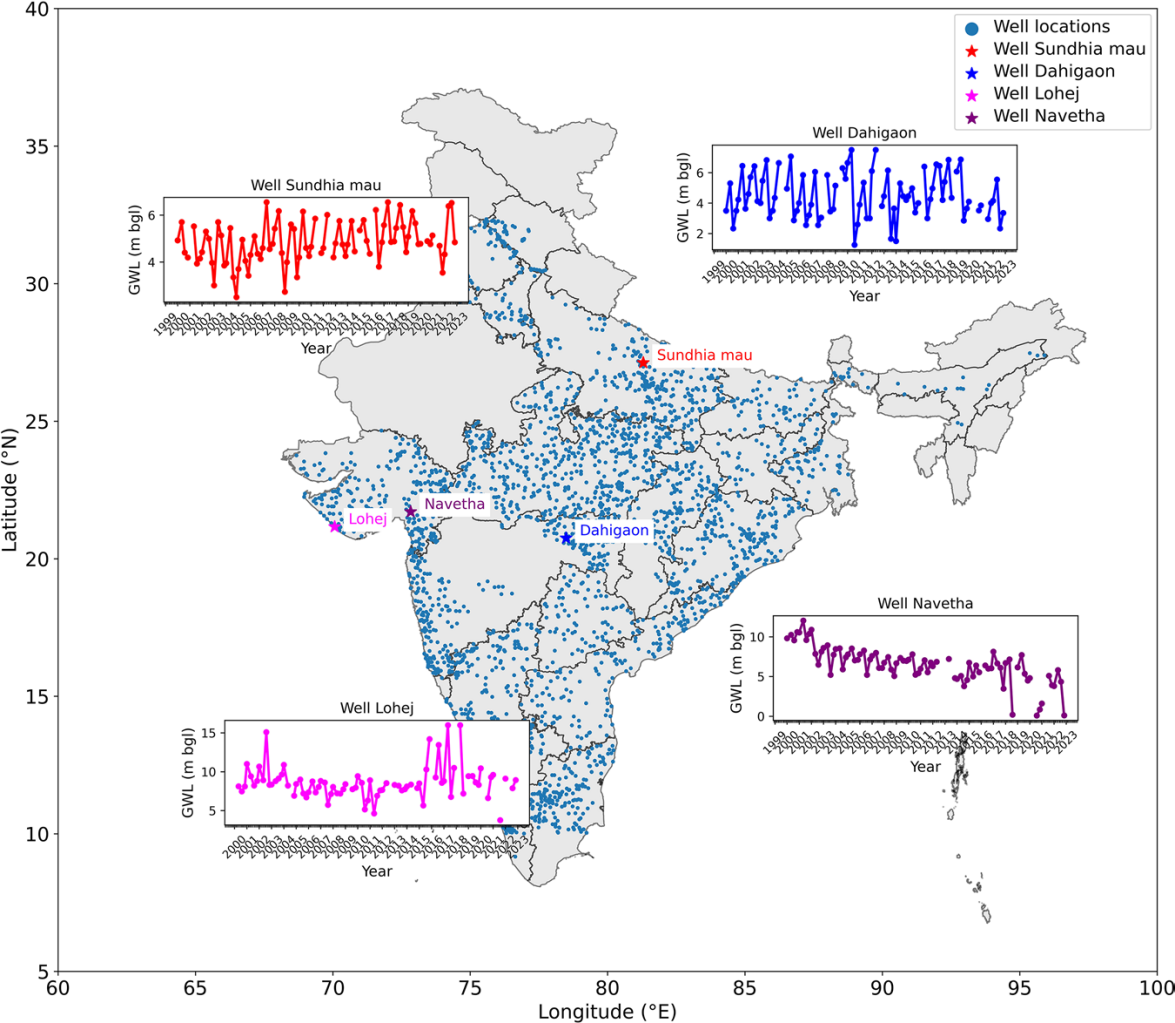
Filtered wells with GWL variations at four random well locations.

To account for natural variations in GWLs over time, rigorous filtering criteria are applied and a final set of 2,759 wells are obtained as shown in Fig. 4. Because of the filtering criteria used, it is evident from Fig. 4 that the 2,759 wells are widely distributed around the nation. We also attempted to perform the analysis using all four values annually for the years 2000 to 2022, which resulted in zero wells nationwide and raises questions about maintenance of seasonal well data by the government. To demonstrate the consistency of the well data with regard to time and space, we included time series of four randomly chosen wells out of 2,759 wells in the same figure.

**Fig. 5:**
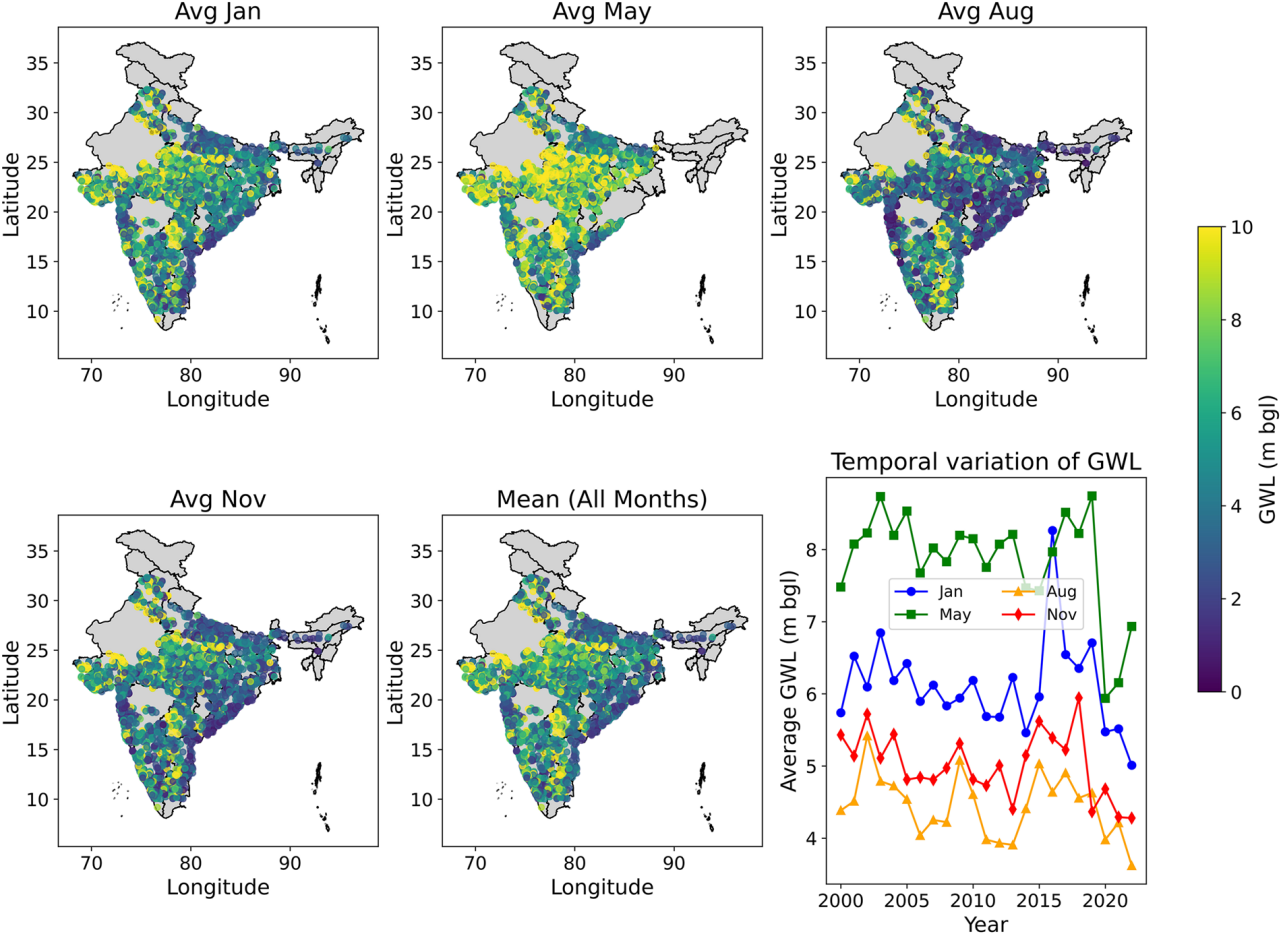
Seasonal GWL variations for filtered wells.

The temporal and spatial distribution of seasonal GWL fluctuations during the months of January, May, August, and November is shown in Fig. 5. Additionally, temporal variation of average GWLs for the months of January, May, August, and November is provided in the final subplot to showcase the GWL variations with time. Given that May represents India’s summer season, when GWL pumping is at its highest, it is evident from the spatial maps that GWLs are lower during this month going up to 10 m bgl. Since the south-west monsoon season (Jun to Sep) brings rainfall to 75% of India, you can see an increase in GWLs during August. As the southern part of India (Tamil Nadu) receives rainfall in the post monsoon season (Oct to Nov), there is an increase of GWLs in the November month spatial plot. In the overall average GWL plot, more GWL deductions can be observed in and around the states of Rajasthan. From the temporal plot, due to increased groundwater extraction during the summer season, May exhibits the largest decrease in GWLs followed by January. Since the south-west monsoon season replenishes all the nation’s surface water and groundwater bodies, August exhibits a good increase in the GWLs. Additionally, you can see a consistent decline in GWLs in January and May between 2000 to 2022, which indicates increased groundwater extraction as a result of unequal rainfall distribution and more groundwater usage for agricultural, domestic and industrial purposes.

### Trend analysis of filtered GWLs across India

**Fig. 6:**
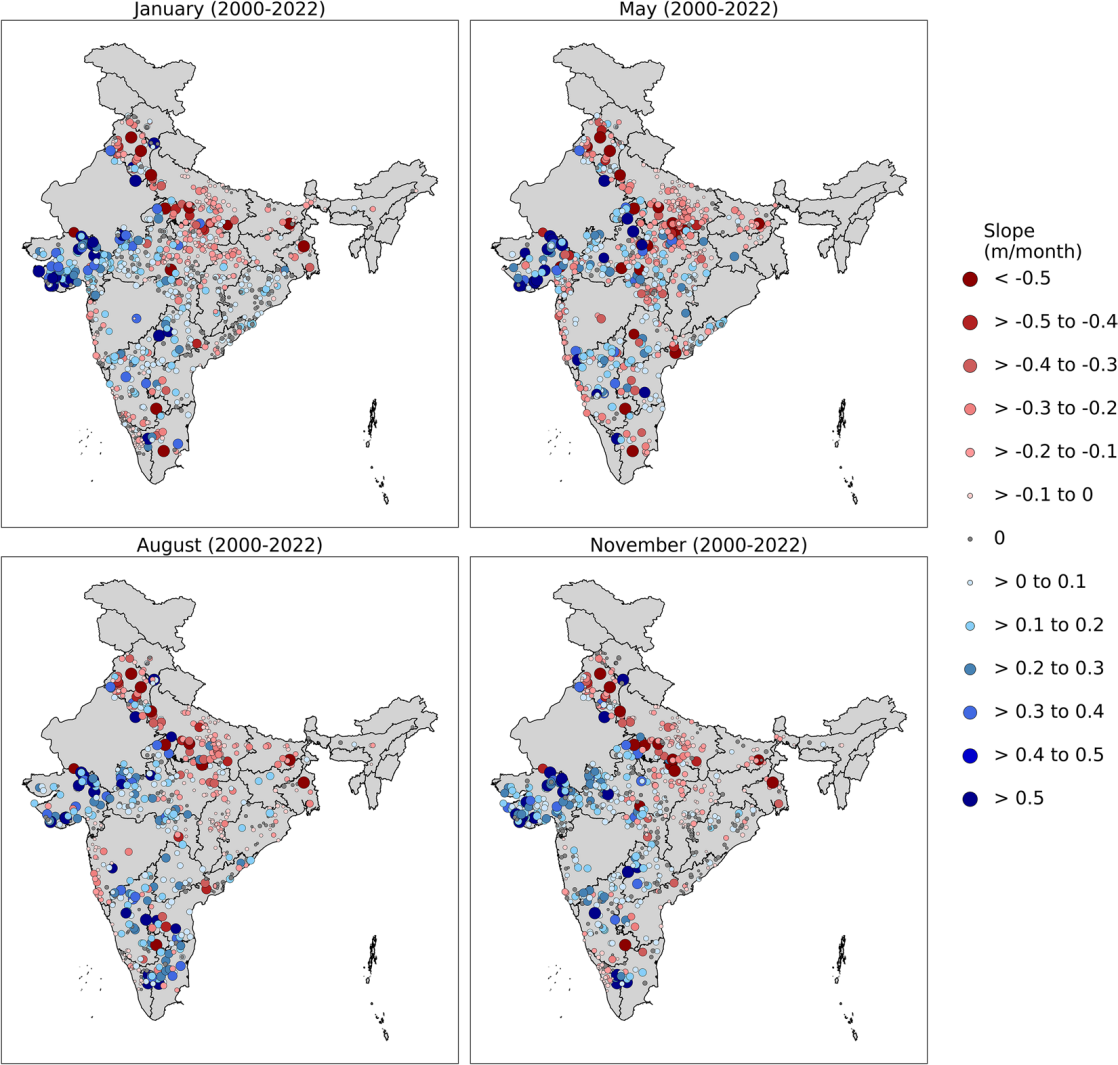
Map of Mann-Kendall trend test (significant at p ≤ 0.05) of filtered GWLs for January, May, August and November months over India for the period between 2000 to 2022.

To assess long-term changes and identify potential anomalies due to data quality issues or changes in measurement protocols, the Mann-Kendall trend test is imposed on each well across India. This analysis is performed at a seasonal scale for the period 2000 to 2022. Figure 6 illustrates the spatial distribution of statistically significant seasonal trends (p ≤ 0.05) across India. Most of the wells exhibit groundwater trends ranging between –0.5 to 0.5 m/month. A prominent negative trend, indicating a decline in GWLs, is consistently observed across all seasons in the northern states, including Punjab, Haryana, Uttar Pradesh, Bihar, and West Bengal. This alarming depletion aligns with findings reported in previous studies^23–25^. Conversely, the most significant positive trends (rising GWLs) are observed in Gujarat and the western regions of Madhya Pradesh. However, season-wise trend variation is observed in Telangana. In the month of May (Summer) a negative trend is observed due to excess withdrawal of groundwater and a positive trend in the month of August (Monsoon) owing to replenishing GWLs.

### Consistency of quality controlled GWL trends across neighboring points

The spatial trend map (Fig. 6) is used to evaluate the consistency of quality controlled GWLs across neighboring locations. The analysis reveals coherent spatial patterns, indicating reliable data quality. For example, wells located in Punjab, Haryana, and Uttar Pradesh exhibit a uniform declining trend in GWLs, while those in Gujarat and the western part of Madhya Pradesh show a consistent rising trend. These spatially aligned trends suggest that the quality-controlled GWL data used in this study are consistent and reliable across adjacent monitoring points.

### Specific yield over India at filtered well locations

**Fig. 7:**
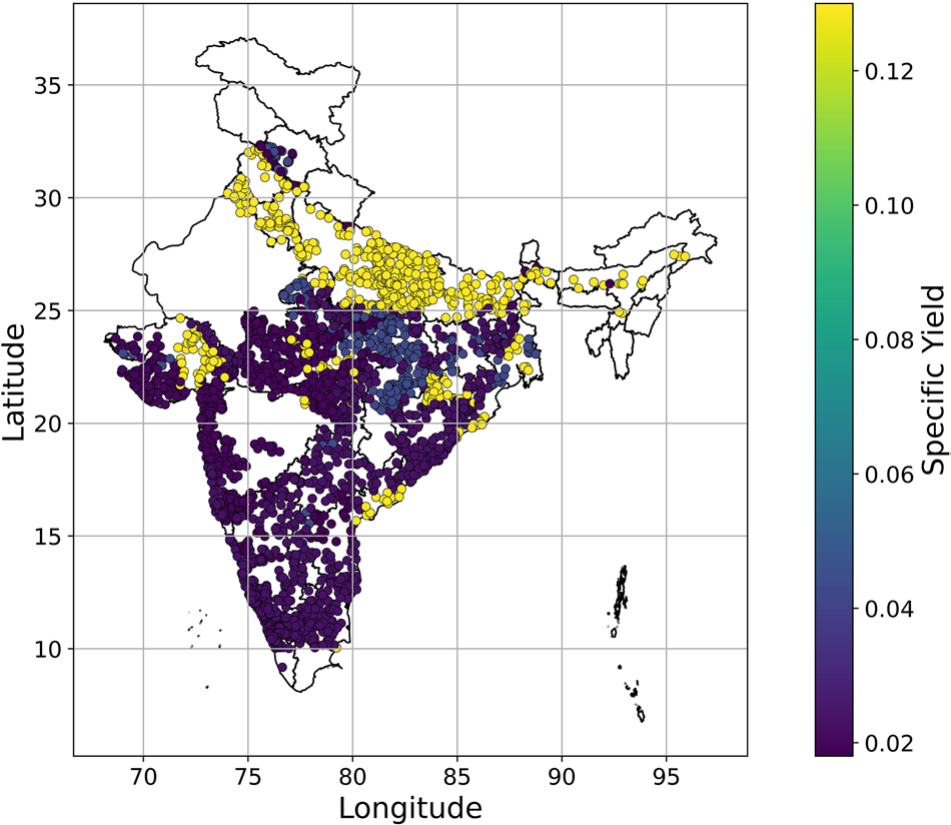
Specific yield map for filtered well locations over India.

The S_y_ of all filtered wells in India is shown in Fig. 7. The map shows good agreement with Fig. 1 i.e., hydrogeological map. Out of 2,759 wells, vectorization-based method successfully classified all wells into five different types of aquifer system as shown in Table 4. As sedimentary and folded metasediment/metamorphic aquifer shows same value of S_y_ which is 0.018. Thus, six different aquifers type reduced to five. Most of the wells (1,165 wells) belonged to fractured crystalline type of aquifer indicating moderate yields (0.023). This type of aquifer was found in peninsular India including Madhya Pradesh. Jointed crystalline aquifer type received second highest well count (625) and found abundant in Gujarat and Maharashtra. This aquifer type corresponds to yield of 0.02. Unconsolidated sedimentary aquifer corresponds to maximum yield of 0.13 and received third highest well count (582), found abundant in north India. The least number of wells are classified into sedimentary aquitards and folded metasediments/metamorphic aquifer. The yield value (0.018) is also the lowest for this type of aquifer.

**Table 4:** Clustered well count in different hydrogeological settings.

S.No	Hydrogeology	S_y_ range	Mean S_y_	No. of wells
1	Unconsolidated sedimentary	0.06 to 0.20	0.130	582
2	Consolidated, permeable sedimentary	0 to 0.08	0.043	256
3	Sedimentary aquitards	0 to 0.03	0.018	131
4	Folded metasediments/metamorphics	0 to 0.03	0.018	
5	Jointed crystalline	0.01 to 0.03	0.020	625
6	Fractured crystalline	0 to 0.04	0.023	1165

### Validation of estimated S_y_ with literature-based S_y_

The S_y_ values estimated using the vectorization-based method are validated against literature-based S_y_ values, as presented in Table 5. For all aquifer types (except sedimentary aquitards) the estimated S_y_ values are consistent with those reported in the literature and fall within the corresponding uncertainty ranges. This agreement supports the reliability of the vectorization-based approach employed in this study.

**Table 5:** Estimated S_y_ and literature-based S_y_ for different hydrogeological settings.

S. No.	Aquifer	Vectorization-based S_y_ (uncertainty)	Literature-based S_y_ range	Reference
1	Unconsolidated sedimentary aquifers	0.13 (0.06 to 0.2)	0.04–0.2	^26^
			0.04–0.2	^27^
			0.04–0.23	^28^
			0.09–0.23	^29^
2	Consolidated, preamble sedimentary aquifers	0.043 (0 to 0.08)	0.002–0.038	^30^
			0.03–0.10	^31^
3	Sedimentary aquitards	0.018 (0 to 0.03)	0–0.08	^26^
			0.06–0.1	^32^
4	Folded metasediments /metamorphic aquifers	0.018 (0 to 0.03)	0.005–0.015	^33^
5	Joined crystalline aquifers	0.02 (0.01 to 0.03)	0.0019–0.0173	^34^
			Up to 0.03	^31^
6	Fractured crystalline	0.023 (0 to 0.04)	0–0.04	^26^
			0.003–0.05	^35^
			0.011 to 0.073	^36^

### Validation of GWSC_insitu_ against GWSC_GRACE_

**Fig. 8:**
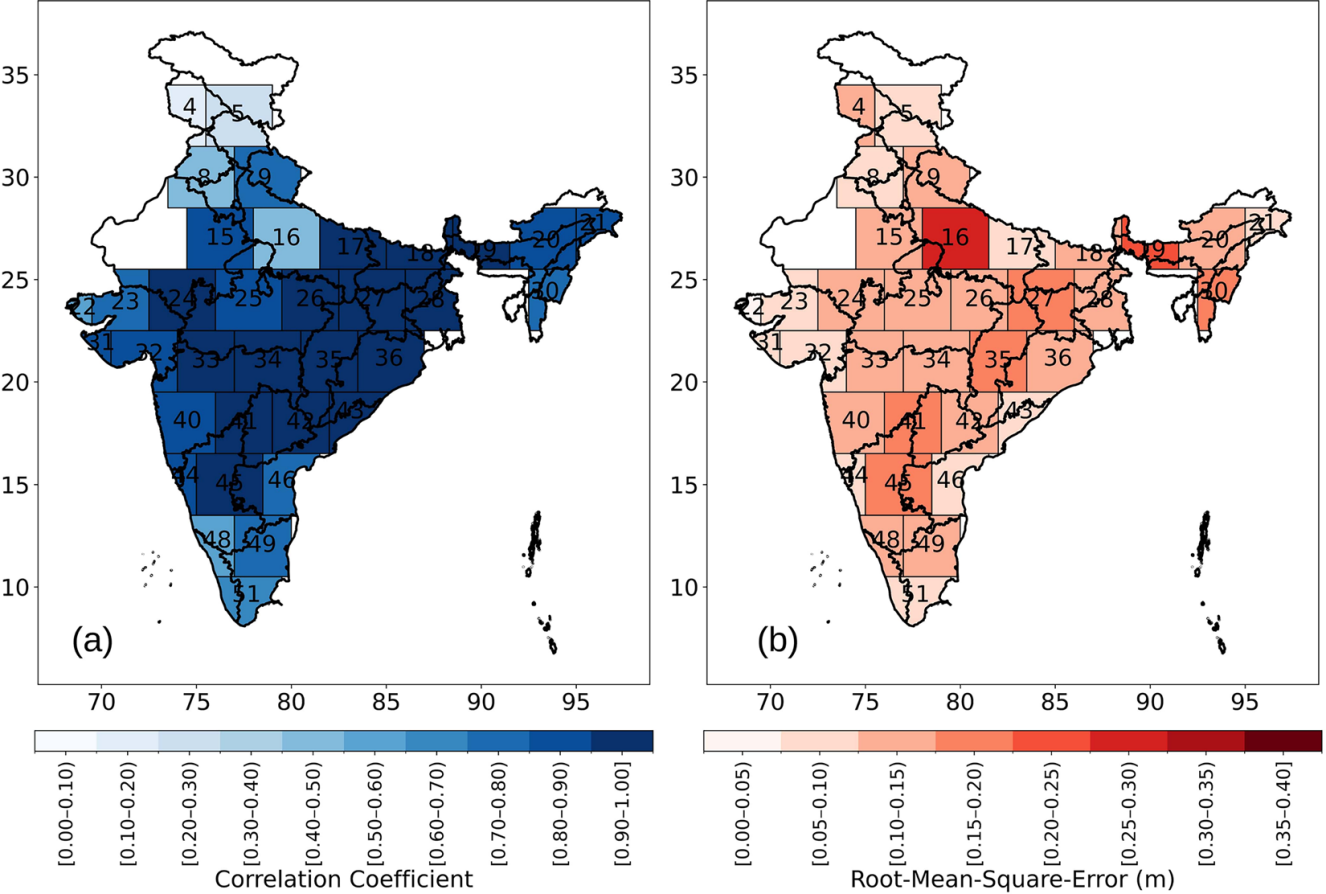
Spatial plot of r and RMSE between GWSC_insitu_ and GWSC_GRACE_ over India. Boxes in the figure shows mascon boundaries and numbers indicate mascon number.

Figure 8 demonstrates the spatial plot of r and RMSE between GWSC_insitu_ and GWSC_GRACE_ over India. Out of the total 52 mascons which covers the whole India only 35 mascons are displayed in the figure where quality controlled GWLs are available. Figure 8a shows that r values are ≥ 0.9 for 15 mascons out of 35. For 9 mascons r is varying between 0.8 and 0.9. Whereas only 2 mascons (4 and 5) showed r value < 0.4. Figure 8b shows that overall RMSE varies between 0.02 m to 0.27 m. Only one mascon (mascon 16) showed RMSE > 0.25 m whereas the rest 34 mascons showed RMSE < 0.25. These results clearly demonstrate that the dataset effectively captures the groundwater signal represented by the GRACE observations.

## Usage Notes

The scripts and the output data are available for download. The datasets for unfiltered GWLs should be prepared by the user following the instructions in the ReadMe file provided along with script and dataset. Then run the python script “1_filteration_code.ipynb” following the instructions in ReadMe file. Finally, quality-controlled GWL dataset from 2000 to 2022 along with S_y_ values is obtained.

## Data Availability

We have used python for processing the data. Along with the quality controlled well data and reference S_y_, we also provide the script freely available for download from figshare link 10.6084/m9.figshare.29293877.v3.
